# An Account of the Good Effects of Opium in a Case of Retention of Urine

**Published:** 1793

**Authors:** Alexander Mather

**Affiliations:** Surgeon at York.


VI. *An Account of the good Effects of Opium in a Cafe of retention of Urine. By Mr.* Alexander Mather, *Surgeon at York. Communicated in a Letter to Mr.* John Pearfon, *Surgeon of the Lock Hofpital and Public Difpenfary, in London; and by him to Dr.* Simmons.

*To Mr.* PEARSON.

SIR,

THE mode of treatment adopted in the following cafe, was fuggefted by the fuccefsful treatment of an affection of the fame kind of which you have given an account in the fixth volume of Medical Obfervations and Inquiries; I therefore think it right to transmit to you an account of the case, as it affords another striking instance of the good effects of opium in cases of retention of urine from a spasmodic constriction of the *sphincter vesicæ*. If you think it worthy of being communicated to the public, as a supplement to your valuable paper, it is much at your service.

I am, &c.

ALEXANDER MATHER.

*York,*

*September* 8*th*, 1792.

### CASE.

Mr. D——, of B——, who had formerly been subject more than once to gonorrhœa, being on a visit to a friend about seven miles from York, in the evening of a Sunday in the beginning of June, 1791, was seized with a frequent inclination to make water, accompanied at first with a slight degree of straining and pain about the neck of the bladder. In a little time the motions became more frequent, and the straining and pain greater; very little urine passed, and about six in the evening he could not part with a drop, and the ſtraining and pain were very violent. Having had two attacks of this kind before, one of which had continued thirty-two hours, and remembering that opium had then been given him to the amount of ſixty drops, he took twenty-five drops of laudanum, and ſoon after fifteen more, and ſent for me.—I took with me three grains of extract of opium.

When I arrived, about eight o'clock in the evening, I found him, every four or five minutes, ſeized with a dreadful ſpaſm, occaſioning an involuntary and ineffectual effort to make water: the agony and ſtraining reſembled much the pains of a woman in labour. I gave him immediately a grain of opium and threw up an emollient clyſter, and directed him to ſit over the ſteam of water, as means that were moſt readily obtained. He ſeemed at firſt to be relieved, but the ſpaſms afterwards returned with greater violence than before. At this time there was no conſiderable tenſion of the bladder, and as every circumſtance indicated the diſeaſe to be purely ſpaſmodic, I did not think proper to attempt to uſe the catheter, leſt the evil ſhould be increaſed. The practicability of introducing it into the bladder was alſo very dubious; for it was with the greateſt difficulty that the pipe uſed ufed for the clyfter could be paffed into the rectum; and during its ftay there the fpafms recurred with greater violence. For thefe reafons, I determined to depend entirely on opium, and to give it in fuch quantity, and at fuch intervals, as the urgency of the cafe feemed to require, *i. e.* till the contractile power of the *fphincter veficæ* fhould be fufpended: I therefore gave him within the hour another grain of opium, and defired that fifteen or twenty drops of laudanum fhould be adminiftered every hour, or, if the violence of the fpafms fhould continue, every half hour, and requefted to be informed in the courfe of the night if he was not relieved.

At two o'clock in the morning I received information that he was no better. I immediately went to him, and, arriving about three, I found he had infenfibly parted with a little urine, but had not flept at all. The plan I had recommended had been diligently purfued.

As the fpafms ftill recurred, though more flightly than when I faw him before, I gave him another grain of opium. After this he fell afleep, and continued dozing near an hour, during which time about a pint and a half of urine infenfibly flowed from him. When he firft fell afleep the fpafms left him and returned no no more; but though he did not ſleep much after the firſt hour, his urine continued to flow without his knowledge, and equally ſo whether he was aſleep or not. Nine hours elapſed after the complaint became violent before he found relief, and in that time he took three grains of extract of opium, and ſeventy-five drops of tincture of opium, prepared according to the London Pharmacopœia. The opium, though given thus freely, left no ſtupor or even drowſineſs. He had leſs ſleep than uſual in the enſuing twenty hours, and his pulſe was not at all affected till after the laſt grain of opium was given; and then it only became ſofter and weaker than natural, and remained ſo during the enſuing day. Forty drops more of the tincture of opium were given in the courſe of the next day and night by way of prevention, and this quantity kept up rather a copious perſpiration while the patient was in bed. When he roſe the next morning (Tueſday) he was as well as he had been before this attack, if we except a very ſlight degree of languor.

The effect of the opium in this inſtance was equally deciſive and pleaſing; and I am inclined to think that the complaint would have given way ſtill ſooner if the medicine had been given more more liberally at firſt. But till we have more experiments to aſcertain the quantity of opium that may with ſafety be given in ſpaſmodic diſeaſes, it will perhaps be moſt prudent to exhibit it at firſt, in ſmall doſes and at ſhort intervals. I think it right to remark, however, that from the inſtances of ſuch affections in various parts of the body that have come under my obſervation, I have learned that opium may be given to ſuſpend ſpaſm in abundantly more liberal quantities without producing its uſual narcotic effects, than in any other diſeaſe in which it is adminiſtered.

